# Mapping services at two Nairobi County primary health facilities: identifying challenges and opportunities in integrated mental health care as a Universal Health Coverage (UHC) priority

**DOI:** 10.1186/s12991-021-00359-x

**Published:** 2021-08-17

**Authors:** Manasi Kumar, Vincent Nyongesa, Martha Kagoya, Byamah B. Mutamba, Beatrice Amugune, Neha S. Krishnam, Grace Nduku Wambua, Inge Petersen, Onesmus Gachuno, Shekhar Saxena

**Affiliations:** 1 Department of Psychiatry, University of Nairobi, Nairobi, 00100 (47074) Kenya; 2 Butabika Hospital/YouBelong Uganda, Kampala, Uganda; 3 School of Pharmacy, University of Nairobi, Nairobi, Kenya; 4 School of Public Health, University of Washington, Seattle, USA; 5 Department of Clinical, Neuro and Developmental Psychology, Amsterdam Public Health Research Institute, Vrije Universiteit Amsterdam, Amsterdam, The Netherlands; 6 Centre for Rural Health, University of Kwa-Zulu Natal, Durban, South Africa; 7 Department of Obstetrics and Gynecology, College of Health Sciences, University of Nairobi, Nairobi, Kenya; 8 Department of Global Health and Population, Harvard T. Chan School, Boston, USA

**Keywords:** Universal Health Coverage, WHO-AIMS, Primary health care, Mapping health services, Facilitators, Challenges, Integrated care model, Mental health integration, Peri-partum depression

## Abstract

**Introduction:**

There is a need to scale-up mental health service provision in primary health care. The current extent of integration of mental health in primary care is pertinent to promoting and augmenting mental health at this level. We describe a facility mapping exercise conducted in two low-income/primary health facilities in Kenya to identify existing barriers and facilitators in the delivery of mental health services in general and specifically for peripartum adolescents in primary health care as well as available service resources, cadres, and developmental partners on the ground.

**Method and measures:**

This study utilized a qualitative evidence synthesis through mapping facility-level services and key-stakeholder interviews. Services-related data were collected from two facility in-charges using the Nairobi City County Human Resource Health Strategy record forms. Additionally, we conducted 10 key informant interviews (KIIs) with clinical officers (Clinicians at diploma level), Nurses, Community Health Assistants (CHAs), Prevention of Mother-to-child Transmission of HIV Mentor Mothers (PMTCTMs), around both general and adolescent mental health as well as psychosocial services they offered. Using the World Health Organization Assessments Instrument for Mental Health Systems (WHO-AIMS) as a guideline for the interview, all KII questions were structured to identify the extent of mental health integration in primary health care services. Interview transcripts were then systematically analyzed for common themes and discussed by the first three authors to eliminate discrepancies.

**Results:**

Our findings show that health care services centered around physical health were offered daily while the mental health services were still vertical, offered weekly through specialist services by the Ministry of Health directly or non-governmental partners. Despite health care workers being aware of the urgent need to integrate mental health services into routine care, they expressed limited knowledge about mental disorders and reported paucity of trained mental health personnel in these sites. Significantly, more funding and resources are needed to provide mental health services, as well as the need for training of general health care providers in the identification and treatment of mental disorders. Our stakeholders underscored the urgency of integrating mental health treatment, prevention, and well-being promotive activities targeting adolescents especially peripartum adolescent girls.

**Conclusion:**

There is a need for further refining of the integrated care model in mental health services and targeted capacity-building for health care providers to deliver quality services.

## Background

The overall burden of mental illnesses in SSA and other LMICs is on the rise. In 2010, mental and substance use disorders were the leading causes of years lived with disabilities (YLDs) in sub-Saharan Africa (SSA) with a total burden of 18.94% [1], and projected to rise from 20 million to 45 million YLDs by 2050 [2]. Previous publications such as the 1990 Global Burden of Disease (GBD) review have also underscored the rising burden of mental illness in SSA and global LMIC contexts [3]. LMICs are poorly equipped to deal with this rising burden. Resources are scarce and inadequately distributed [4, 5], and mental disorders receive as little as 1.6% of LMIC government health budgets and 0.4% development assistance for health [6, 7].

In Kenya specifically, mental illness accounts for 4% of significant health conditions—commensurate with the global burden of disease estimates for mental disorders [8, 9]. As is the case elsewhere, the mental illness disease burden is on the rise [10] and there is a large mental health treatment gap, with a low budgetary allocation to mental health and a deficiency of trained mental health professionals [11]. US Institute of Medicine in their workshop proceedings on integrated mental health in SSA published in 2016 [10], highlighted that Kenya, with a total population of 47 million people [12], has less than 500 trained mental health professionals comprising only one hundred psychiatrists, the majority of whom were based in Nairobi, the capital city and 12 neurologists mainly working in private hospitals in urban settings [10, 13] and about 2100 psychologists and over 100 psychiatric nurses (email communication by an official of Kenya Psychological Association, 2021 June). A study carried out in 2015 in the coastal county of Kilifi, one of Kenya’s 47 counties, established that there were no psychiatrists or psychologists in the county, with the two psychiatric nurses in service expected to serve a population of 1.2 million people for example [14]. Poor supply of services is matched with poor demand and access. In addition, widespread beliefs that mental illness is a result of witchcraft alters health-seeking behavior [15]; stigma, discrimination, lack of accurate information about mental health conditions, where to seek services, and economic challenges make it difficult for patients to afford consultation, medication, and rehabilitative services are some of the barriers to access mental health services [16].

The World Health Report [17] urges country-level investment in research and policy advancement to globally improve health services coverage, with prioritization of support and strengthening of primary health care services [18]. To this end, the integration of mental health into primary health care using a task sharing approach is encouraged. An important step in order to make a case for better and holistic integration is to map existing service structure and practices to know how many (if at all) and what quality mental health services are being offered on ground. The World Health Organization’s Assessment Instrument for Mental Health Systems (WHO-AIMS) [11] was developed explicitly for LMICs to evaluate crucial aspects of mental health systems through established routine monitoring systems [19, 20]. WHO-AIMS enables assessing the mental health systems, delivery, and support required for the vulnerable populations in primary health care [20]. This strategy is supported by the WHO Mental Health Gap Action Programme (mhGAP) which aims to provide health planners, policy-makers, and donors with a set of clear and coherent activities and programs for scaling up care for mental disorders. The program created a Framework for Country Action, which provided countries with recommended steps to scale-up their interventions and treatment for mental, neurological, and substance abuse disorders [21–23].

Following these steps, the aim of this study was to conduct a situational analysis of the extent of mental health integration in health centers in Nairobi County in Kenya and develop an understanding of facility-level barriers and facilitators to the integration of mental health into routine care for adolescents. Adolescents and young people dominate Kenyan population demographics and there is evidence that mental disorders are on rise in this demographic [24–26]. Adolescence is a critical transitional stage in life, and many adolescents experience challenges such as independence, self-identity, family dysfunction, which all contribute to mental illness. Providing youth-friendly services is challenging due to developing physical, emotional, and mental health that requires specifically tailored interventions [27, 28]. It is crucial to identify gaps in existing services and evaluate the effectiveness of this targeted population.

## Methods

### Study area

**Fig. 1:**
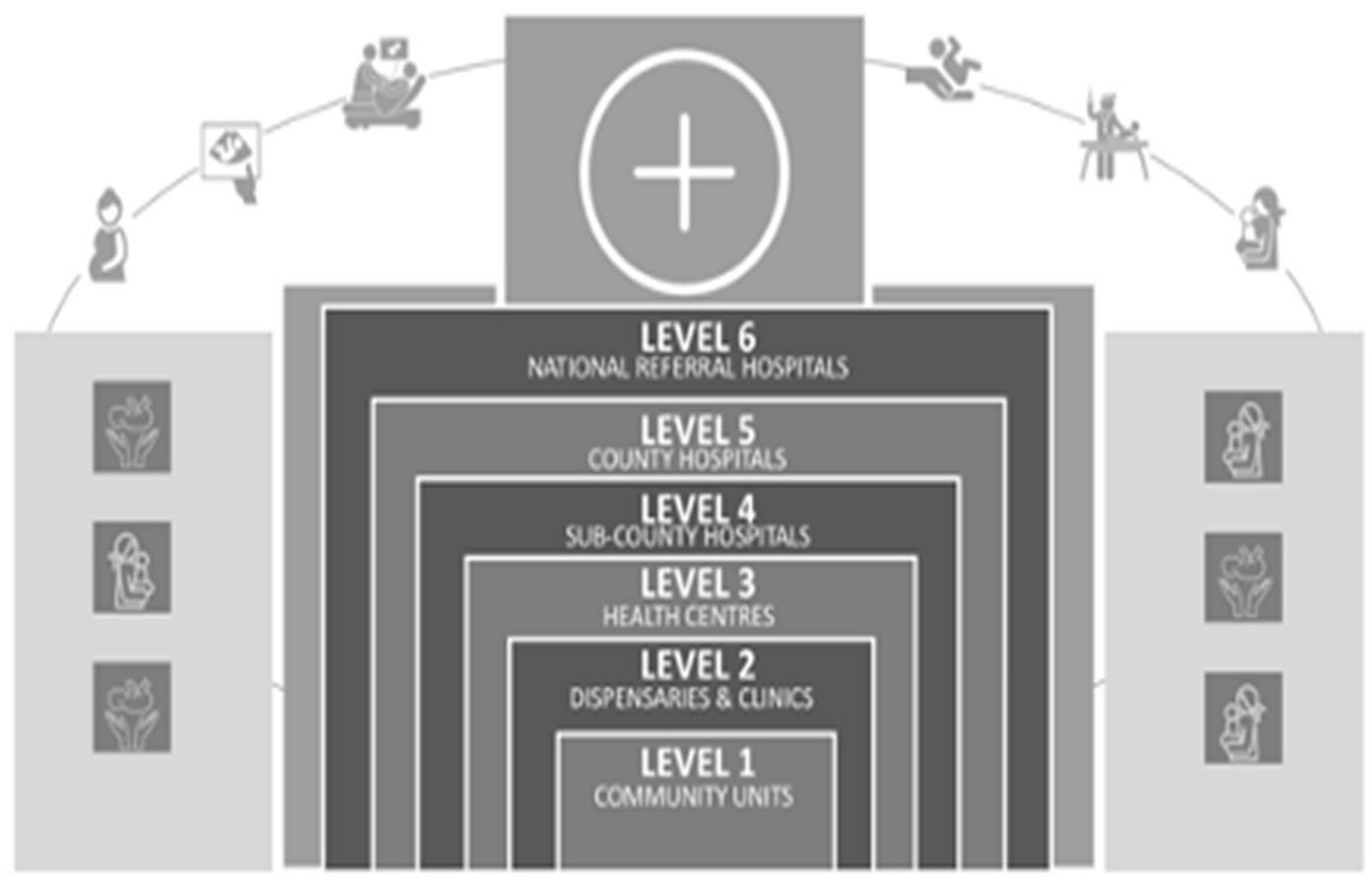
Healthcare structure in Kenya

The study was carried out in two health centers in Nairobi County, Kenya’s capital city. Through the Kenya Essential Package for Health (KEPH) [29], the health care system is divided into six levels that include*:* Level 1 Community Units; Level 2 Dispensaries and Clinics; Level 3 Health Centers; Level 4 Sub-County Hospitals; Level 5 County Hospital; Level 6 National Referral Hospitals (see Fig. 1). In Nairobi County, the public health workforce is estimated to have 3290 personnel, of which 2604 (79%) are technical staff. The technical staff mainly comprise General Doctors and Specialists, nurses, clinical officers, public health officers, and laboratory technologists/technicians [30].

The Nairobi County health sector handles all the County health infrastructure. This includes preventive, curative and protective, reproductive health services through a network of over 80 health centers and three hospitals spread across the city. It covers—paternity care, public health inspectorate, epidemiology and disease control, Building Occupation Certificate, School Health, Pumwani maternity Hospital, Mortuary and Funerals, Nursing, Ambulance, Nutrition, Inoculation, Training [31].

### Theoretical framework

**Fig. 2:**
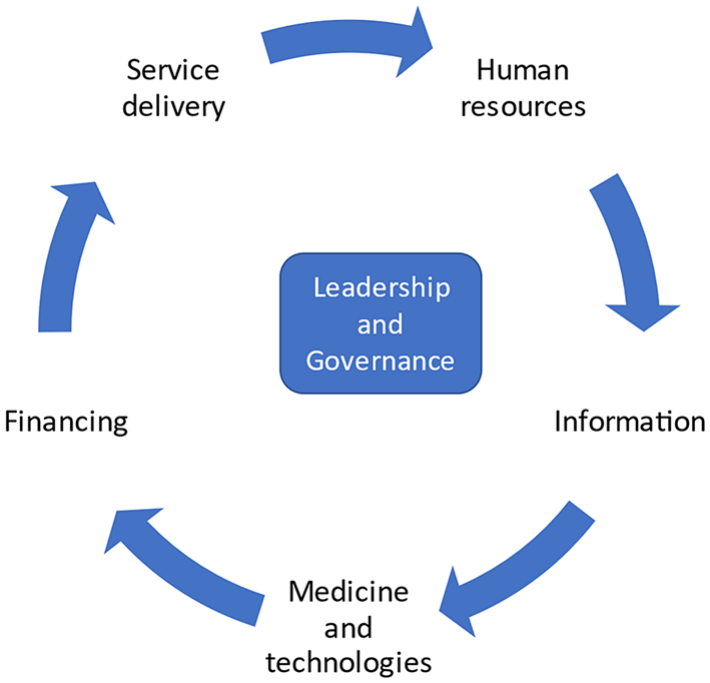
WHO’s mental health systems strengthening approach [32]

A health system is more than the pyramid of publicly owned facilities that deliver personal health services, it comprises organizations, people and actions that maintain or promote health (WHO, 2007). In order for it to provide these functions, it utilizes several building blocks. Service delivery and health care workforce focus on safe, effective, quality personal and non-personal services, and work force is needed to discharge or promote these services. A health information system is needed for production, analysis and dissemination of reliable and timely information on health determinants, health system performance and health status of all groups of the population. Medical technologies generate quality, safe and effective products and services needed for running the health system. Appropriate financing enables the system to fund efficiently but also ensures provisioning of support for vulnerable and needy populations. In the context of mental health services in Kenyan primary care, we have focused on services and human resources mainly (Fig. 2).

### Study design

We used a qualitative approach, to evaluate existing services, cadres, human/financial resources, and funding partners in the two selected health centers in Nairobi County. We made use of WHO-AIMS Framework for country action [33] to design a semi-structured interview guide to capture existing services and integration of mental health services, staff training, and ability to deliver mental health services (see Table 1). The WHO-AIMS domains include: (1) policy and legislative framework; (2) organization and integration of mental health services; (3) mental health in primary care; (4) human resources; (5) public information and links with other sectors, and (6) monitoring and research.

**Table 1:** List of questions and probes on KIIs

1. What are the types of health services offered at this particular facility? How often do they run?
2. Who provides these services to the patients? Level of education?
3. Who are the external partners who help lighten the load? Who are they and what kind of support to they provide?
4. When you think about mental health, do you feel that the facility helps the community in that area?
5. What are some of the barriers and facilitators the facility has in providing mental health services for the population it serves?
6. What do you understand by integrated mental health?
7. What do you think is needed to integrate mental health in PHC? Is it a long-term or short-term goal? Could you explain?
8. In your own role as a nurse/technician/social worker, what adjustments are needed to accommodate mental health services?
9. Do you feel you are equipped to provide services?
10. What would you need to address specific needs for you to offer mental health Services?
11. Do you need—knowledge—please explain what that means?
12. Do you think—attitudes—need changing—please explain what that means?

### Participants

A team of four researchers (MK, BM, MK, VN) carried out facility visits to introduce the work, ascertain services and available staff. To assess personal views on mental health services in primary health care, we enrolled twelve (12) Health Care Workers (HCWs) for this study. The two facility in-charges were selected due to their administrative rank, while the other ten HCWs were invited to participate from different clinics.

### Data collection

The identified HCWs were approached and were presented with an information sheet clearly stipulating the study purpose, procedures, benefits, and risks in which no physical or emotional harm was anticipated. Those who agreed to participate gave written consent prior to the interview. The participants were informed about their rights including the right to withdraw at any time should they need to without any implications to them. They were assured of the confidentiality of their individual response and requested to provide information about services and clinics in their facility and the kinds of challenges they encounter in offering mental health care. No HCW refused to participate. The interviews were conducted by trained researchers (MK and VN) and audio recorded. Each HCW was conveniently and privately interviewed in person from their respective facilities while on duty. Participants were probed about their views on the importance of mental health services in their facilities and their knowledge and exposure to the field of mental health in primary health care services. HCWs included facility in-charges, Antenatal Clinic (ANC) Nurses, Prevention of Mother to Child Transmission (PMTCT) Nurse, child welfare clinic (CWC) nurses, family planning nurse, comprehensive care clinic (CCC) nurse, mentor mother and Community Health Assistant (CHAs). Interviewees were offered transport money as stipulated by the study protocol that covered for their time and offered a token payment. Another data collection approach was a document analysis collated data on facility staffing and clinic numbers and timings. The data were audio-recorded and the interviewing team kept a record of key feedback, significant comments and feedback from the participants.

### Ethics approval

Ethical approval was obtained from the Kenyatta National Hospital and University of Nairobi Ethics and Research Committee (Approval No. P694/09/2018). Permission was obtained from Nairobi County Health Directorate (Approval No. CMO/NRB/OPR/VOL1/2019/04). A research license was also obtained from the National Commission for Science, Technology and Innovation (Approval No. NACOSTI/P/19/77705/28063).

### Data analysis

**Fig. 3:**
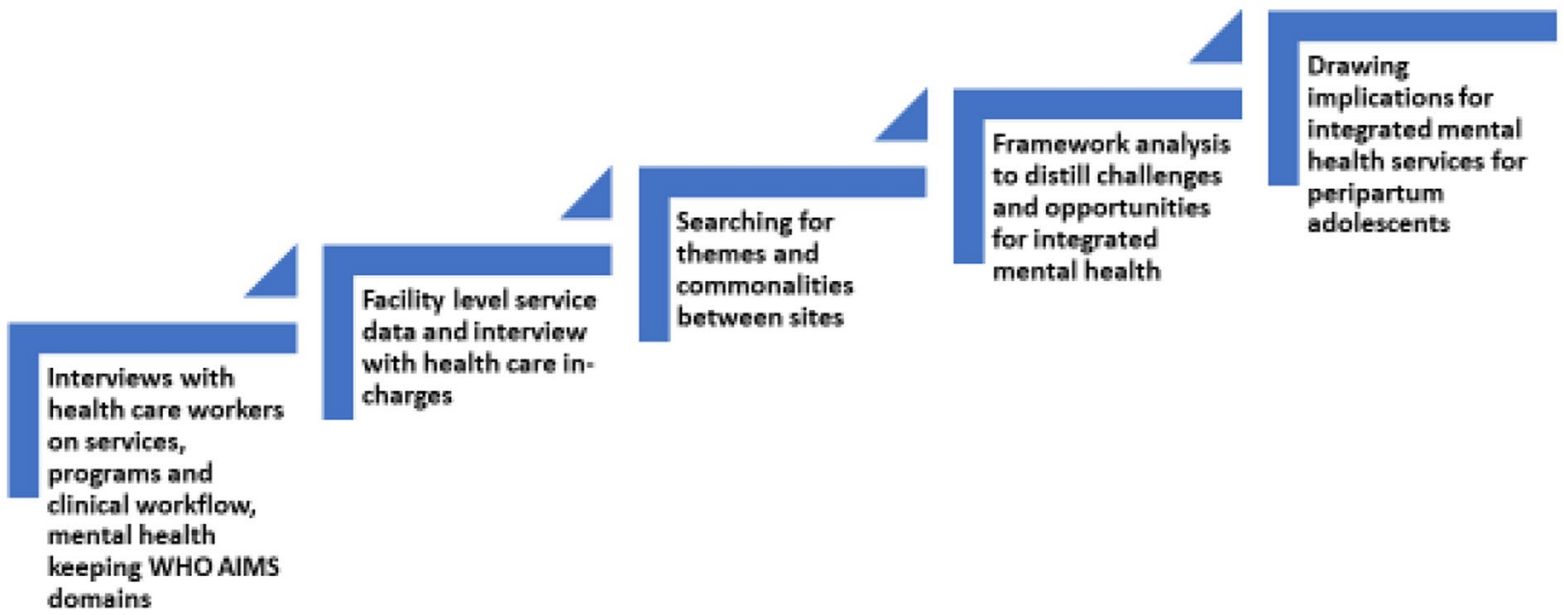
Data collection and analysis process

To determine the barriers and facilitators in delivering mental health services, we utilized a framework analytic approach. Framework analysis is a pragmatic data analytic method which involves five steps of data management such as familiarization; constructing an initial thematic framework; indexing and sorting; reviewing data extracts; and data summary and display, followed by a process of abstraction and interpretation [34]. We looked at the services, cadres, and other partners at the two facilities while identifying the capacity-building needs for mental health integration in primary care health centres (Fig. 3). Audio recordings of interviews were transcribed verbatim, translated from Kiswahili to English, and de-identified to protect their confidentiality prior to analysis. One researcher (M Kumar) from our research team read each transcription and manually coded data into various themes based on interview content (i.e., barriers to integrating mental health services, necessary training, and HCW attitudes). Other researchers in the team performed a secondary review of themes and consulted to resolve any coding conflicts. Results were determined from data from the twelve (12) transcripts. Themes for barriers and facilitators in mental health services are supported by illustrative quotes, as seen below. Participant names and other identifying information have been changed for confidentiality reasons.

## Results

### Study informants

Of the twelve (12) health care workers were enrolled in the study, ten (10) were females and two (2) males. The oldest participant was aged 55 years, while the youngest was 36 years. Half of the participants were nurses six (6), two (2) clinical officers, one (1) psychiatric clinical officer, two (2) community health assistants, and one (1) community-based mentor mother. Eight (8) of the participants were recruited by the Ministry of Health, while three (3) were recruited by external funding partners representing clinics such as Prevention of mother-to-child transmission, antenatal clinic, child welfare clinic, family planning, and psychiatric clinics. This information was confirmed by the health records accessed through the facility in-charges. See Table 2 for more information on services and human resources.

**Table 2:** Characteristics of the two health centers

		Kangemi	Kariobangi
Staff		51	41
Services		TB clinic, antenatal, maternity, family planning, child welfare clinic, comprehensive care clinic, HIV testing, path laboratory, nutrition, immunization, psychiatry clinic, non-communicable diseases clinic	TB clinic, antenatal clinic, family planning, child welfare clinic, comprehensive care clinic, HIV testing, path laboratory, nutrition, immunization, psychiatry clinic, non-communicable diseases clinic
Support	Internal	Ministry of Health, Nairobi County Council	Ministry of Health, Nairobi County Council
	External	Afya Jijini (USAID) Aids Healthcare Foundation (Japan), Malteser, Jhpiego,	Afya Jijini (USAID), AIDS Healthcare Foundation (Japan), Concern Worldwide, MSF (France),
	Liaison psychiatry	Kamili Organization	Mathari National Teaching and Referral Hospital

### Services

In both facilities, clinics were running 5 days a week except for the hospital’s psychiatric clinic once a week. Services are limited, with maternity services being offered only in the Kangemi health center, and youth-friendly services are not offered optimally in either clinic. Despite this gap, these community health facilities cover several conditions. Their services include tuberculosis, antenatal and maternity, family planning, child welfare clinics, Comprehensive Care Clinic and HIV testing (HTS), path laboratory, nutrition, and immunization. We found that three to four clinics ran lower frequency clinics than usual 5 days per week due to staffing issues. For instance, both facilities’ psychiatric clinic is held once a week and managed by outsourced staff from a mental health nursing organization called Kamili Organization. The non-communicable disease (NCD) clinic is held weekly at Kangemi and bi-weekly at Kariobangi Health Center and provides services on managing and preventing diabetes, hypertension, and cancer. While male health services in Kangemi are infrequent, the Kariobangi center offers a dedicated service where some male CHVs help the male patients with their medical requirements. Health care workers reported that they made various efforts to engage with youth by tailoring services providing them with unique slots for sexual and reproductive health and HIV services to offer privacy and stigma-free clinical encounters. Using the clinic proved to be a source of shelter and a haven for many young women and adolescents who were homeless or exposed to violence and abuse as a short-stay option. Despite several attempts at engaging youth, we did not see many well-developed youth-friendly services in either facility.

We found that many of the services were outpatient, running from 0800 to 1700 h except for the maternity services at Kangemi Health Center, which operated throughout (24 h all days of the week). The services are run by clinical officers, nurses, laboratory technicians, while the community health assistants (CHAs) and community health volunteers (CHVs) actively escort patients to these services. There are high numbers of undiagnosed patients and unattended to mental health services from what we learned from our engagement. The numbers that are seen can quickly become a source of strain given that very few health care providers offer these services.

### Financing and human resources

The Kangemi Health center has been receiving external funding from Afya Jijini (USAID), Aids Healthcare Foundation (Japan), Malteser, Jhpiego, Kamili Organization, and the national level support from the Nairobi City County and the Ministry of Health (MOH). The Kariobangi Health center similarly has been receiving external funding support from Afya Jijini (USAID), AIDS Healthcare Foundation (Japan), Concern Worldwide, MSF (France), and the national level support from Nairobi City County and the Ministry of Health. Liaison psychiatry and community mental health expertise are offered from both Mathari National Training and Referral Hospital and Kamili Organization, a local NGO specializing in training nurses in mental health care for outpatients. Many of these services combined integrated sexual and reproductive health services, youth-focused and mental health services.

We offer key barriers and opportunities our participants shared with us around integrated mental health services based on this data.

### Systemic barriers

#### Lack of integrated services

While discussing barriers on a systemic level, participants identified a lack of integrated services as a hindrance to improving mental health outcomes. Staff conveyed various resource and infrastructure challenges such as:

*“- okay like the-the-the integration of family planning you know previously family planning-family planning-we have family planning room in outpatient so our clients we used to send them there so they integration is not a bad thing of family planning it is good since our clients will not queue again you know but the room is not-is not convenient to provide the service like you see we don’t have water, we don’t have sink yes, so hygiene is an issue and we don’t have a couch as you can see for you to give an implant you have to-the patient has to be on bed on a couch so it limits-it limits our capacity to-to provide the service so you see the management will take it as if we are rejecting or we are rejecting the change but it is- it is not-they have to act on those things before we go ahead because we are only giving injection and if you talk to a client and she decides that she wants implant you know you will not convince them that let me give an injection because that is what I can give I told them I will just be sending them until we do one two three things [some silence] but it is a good thing [some silence].”…… “The other policy is for the-the-the CCC and PMTCT to give drugs, pharmacy is incorporated in our [laugh].”* Female Nurse

Another provider described how implementing integrated medical services within healthcare would be vastly significant:

*“Integration is when we incorporate mental health care into all service points like when someone comes to my clinic he/she is able to be screened for mental health and just the same way we are doing with TB as in every work station somewhere even from the gate the soldier can screen and know that this is a person with depression or stress or what and they may be able to assist them in a way or another. So that is the integration that-that mental health that someone-any client is able to be screened for mental health issues at every workstation facility”. Reason “Yes, because previously people used to feel like mental health is-they used to stigmatize the mental health and they used to attach it to people who are mad they are in Mathari but mental health issues start from- as in with us even us the health care workers could be struggling with mental health issues and we transfer them to our clients, so it is very important when from really primary health care everyone is so conscious about their own mental health so that even as they take care of clients, they are sober and they give quality care and just to protect them from those clients from having bigger issues of mental health like depressions.”* Female Nurse

#### Lack of support

Another area of concern was the lack of support for mental health services. Below, participants mention the need for more significant support from partner and donor agencies:

*“it’s my prayer that the partners they have on board may implement because most of these health partners who come on board they are either on a research or maybe they are just passing by for one or two issues then they end the support. It is my prayer that if we may get more of the support and also if they can impart more knowledge to the health providers given the different clinics I think this may be of help, because I may attest as a person that since you came on board I am able to serve my clients better than I was previously. So I would like also maybe the same knowledge to be imparted to the other health providers and we may see ways of sustaining the same just by the knowledge, if you give us a lot of knowledge we may look for ways of sustaining the same services in this clinic and for the benefit of our clients; as I know most of our clients as we interact with them we see that they have issues that really need to be sorted out and I think this is one of the areas where we meet like every other person in the community; here in the health center, yes. But thank you very much for the much that you have done “—*Female HCW

#### Privacy concerns

Many healthcare workers highlighted issues of privacy and feeling that their facility lacks appropriate visual and auditory privacy:

*“Yes. We lack- I think in terms of rooms and because sometimes you just find that the counselor is outside with a client talking so you know even for the-for you to capture the concentration of that client is a challenge, because the client may see somebody whom he/she knows at stares at him/her so and you need a quiet room and also privacy and confidentiality of a client is very important”* Male Nurse

“*We need a room, a permanent room for psychiatric clinic, with all the drugs stored there with a permanent staff there specifically to deal with psychotic patients and psychotic issues”* Male Nurse

### Social barriers

#### Culture and stigma

Participants also talked about social barriers in terms of cultural beliefs and stigma surrounding mental health that need to be urgently addressed. One nurse expressed difficulty addressing mental disorders to a patient with a different cultural belief:

*“Because there are some cultures where people don’t even go to hospital when they are sick; they believe in prayer. So such like when you meet such a patient it will be hard to convince this client to come to the facility because he has a belief that if at all he can be prayed for he will be healed. In attitude you know there are those staffs who normally take that in psychiatry we are dealing with mental issues, it can just be a minor medical issue or a major or something you can handle even not using drugs, something you can come maybe talk with the patient not a must he or she ends up taking drugs …These beliefs you know most of the youths they have that mentality that when you are stressed you end up using drugs that it reduces stress; something like that. When you have family issues maybe with your wife and you have an issue you end up taking drugs or alcohol so that you feel like you are relieved”* Male Nurse,

### Individual barriers

#### Attitudes and behavioral issues in HCWs

The first barrier that health facility workers identified has to do with their jobs. HCWs described that a change in provider attitude and behavior is essential to improving health services. One provider commented on the necessity for change:

*“- we need to change because for example like an adolescent who is having HIV there is still stigma even within the staff members, after the client has come they are like “that girl and the way she is young, where did she get HIV from…”* Female, HCW

Another provider described how she is already changing her approach towards clients to improve trust levels:

*“I must say that to some extent your team has also helped us to realize that some of our attitudes will make the client either to open up or not to open up. So very true and I must say for the last three or so months I am experiencing some changes; now that we have interacted with your team we are able to approach these clients in a better way that will enable them come very close to us as compared to the previous times whereby they could share just a little bit then maybe reserve the major—.”* Female HCW

Other providers shared how good attitudes held by health care workers could positively impact their patients’ outcomes:

*“Yes; there has been a good rapport of late and one of the areas, maybe I can point out one area; we have group sessions and during the group sessions we start ourselves by giving our experiences and given the experiences we also allow them the space to come in and share what they think we can do better to help them and this one has enabled clients to open up especially in the groups and for those who seem to be a little bit reserved we have also given them the space on a one on one interaction and things are working better. So it is a plus, for the last three months yes we are seeing different results from the clients and also from the health providers”* Female HCW

#### Lack of training

Only three participants among the two facilities had psychiatric training, with one participant who had a psychiatric diploma. The remaining HCWs reported being exposed to mental health topics back in-school training; yet, all participants equivocally shared that their previous exposure was limited. Below, a nurse describes her work setup and desire to receive further mental health exposure:

*‘We would love to learn more about mental health. Basically, most of the people have gone through; like for the nurses and clinical officers they have gone through the basic training on psychiatry. The normal; the basics but there is more into the diagnosis—so when it comes to knowledge people just; people are not very well acquainted with how to identify early. Mostly we only think the psychiatric people are people who have reached a point where already they are maybe talking to themselves; you can see the psychiatry condition physically. But we are not yet at that level of identifying the psychiatric condition just by interviewing”* Female Nurse

A common theme regarding the necessity for more exposure and better education surrounding mental health was stressed throughout various interviews. One nurse describes the importance of integration of services and proper mental health training:

*“To integrate the mental health [Silence] I think it is training of staff so that when for example it’s on a Friday and a mental health client comes; I am able to do the counseling, I am able to cater for his problems and in the process if I find that he has another problem I take care of it”* Female nurse

Other participants discussed learning new skills that could help them engage with their clients better. Providers echoed a shared struggle to get their clients to open up due to communication barriers that impacted help-seeking behaviors. Below, providers talk about the benefits of having an orientation or classes concerning their role:

*“I may say to some extent yes I’ve been given some information though I may not say I am fully backed for the position I believe given a chance probably some orientation or maybe a class or two I may be more empowered to handle the clients, but tentatively basically the knowledge I’ve acquired from your team I believe at this juncture I may be able to look at a client and tell that this client is not in the same mood probably like I saw them in the previous session. I may be able to detect one or two things that may be an indicator that this client needs some mental health care session.”* Female HCW

Another participant emphasized adequate training would be able to provide staff with the confidence of providing hands-on support and timely care:

*“Okay, maybe just basically I would say refresh a bit and also some little empowerment on the same because there are those who get a little bit reserved so you may not be able to take it out from them. There are those who are a bit adamant; maybe the skill of how to go about it I believe I may need to be orientated on that to be able to get through to this client to open up”* Female HCW

## Discussion

Guided by the WHO’s Building Blocks approach [35] the study aimed to map the available health services, assess existing mental health services, human resources available in the primary health care centers. Additionally, in this mapping exercise we try to identify factors impacting integrating mental health into routine care with particular attention to adolescents. As shown in Table 2  and Fig. 3, we found that each health facility provided several free health services, daily outpatient services, weekly mental health services, and weekly clinics (such as non-communicable clinics). All health facility participants we talked to did not know about mental health policy or legislature including standard facility based or public information protocols for mental health care. They were also unaware of public information on mental health care for dissemination, and links with other sectors, including public health monitoring and referrals when asked about these during the interviews. Through in-depth interviews and service data, we identified a significant gap in the staff training around mental health and psychosocial support. This training gap may explain how our interviewees were unsure of the quality and evidence-informed mental health services to patients that they see. We learned from our participants that they had received brief training during their nursing or undergraduate medicine training or internship on some mental health concepts, which needed further strengthening. There was an urgent need to integrate this training with a focus on mental health needs of vulnerable and key populations that these county clinics target such as women and young mothers living with HIV, pregnant and parenting adolescents, LGBTIQ youth, men living with HIV, individuals with disabilities and children with developmental delays or disorders. The lack of staff training poses a challenge since clinical officers and nurses are the first contacts for clients and adolescents. Findings from a study conducted in Zambia among primary health care workers showed that health care workers noted increased training as the key to integrating mental health in primary care for early identification and management of mental disorders [5]. The dearth of knowledge and clinical procedures limits screening, diagnosis, treatment, referral of patients was also noted in our interviews by the study team. This is an important finding necessitating further follow-up and investigation in other clinics too. We also found that burnout was reported due to the shortage of facility staff, yet the patient turn-up in the facilities is high. Our findings demonstrate a need to increase human resources capacity and the health system’s capacity to achieve the Kenyan targets for preventive and promotive mental health services. Similar findings were recorded in a study carried out by Jenkins et al*.* [15] on health systems challenges to integrating mental health delivery in primary care in Kenya. Their findings recorded barriers such as workload and shortage of trained staff [15]. Clinics must be adequately staffed to implement mental health services in PHC, with trained professionals to identify early, diagnose and treat health problems. We know that lack of proper training limits the number of patients that clinics can provide care to and may not help patients receive adequate support in time. We think that though our study was a small qualitative evaluation study, we did capture important gaps and barriers that are experienced by HCWs in delivering quality mental health services in primary care. We do not know any other study that has tried to review such gaps in recent mental health literature focusing on Kenyan primary health care.

**Fig. 4:**
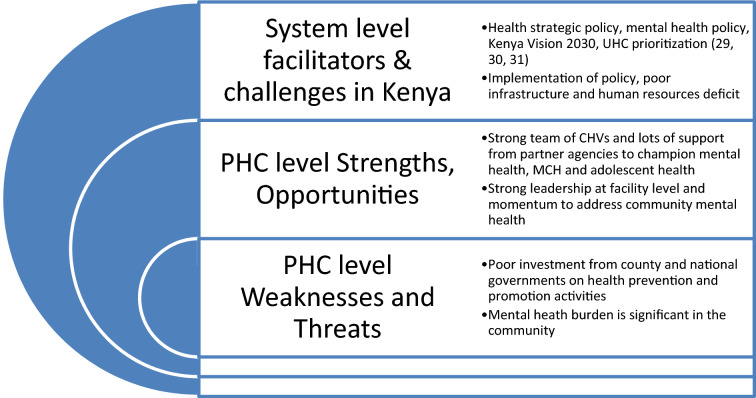
Mapping of facility-level resources and services for integrated mental health care

See Fig. 4 which summarizes barriers around mental health system and services in primary care in the two settings we focused on. Health systems strengthening is achieved through an iterative development and bolstering of these six elements of the building blocks. An investment in multiple and dynamic partnerships within government, county health structures, with private sector as well as civil society including users of these services is critical. Such partnerships strengthen accountability and quality, equitable services. Petersen et al. have in their multi-country work underscored that leveraging existing health system processes that are synergistic with chronic care services and strengthening healthcare system building blocks to provide a more enabling context for mental health integration are important and this process goes beyond trainings of health care workers or introducing new patient care interventions [36]. Even though our participants were well aware of the need to integrate mental health services into routine care and how challenging integration is as an exercise, they expressed limited knowledge about mental health disorders. We found several individual levels as well as health system barriers that came in the way of integrated and collaborative care. Our finding is akin to a study carried out in South-west Ethiopia among primary health care workers [37] where similar lack of holistic investment in health systems was a primary cause of.

Barriers we identified included lack of time, space, and resources to roll out quality mental health services, lack of mental health trained personnel, and limited funding to promote mental health education and integrate mental health services within healthcare. Most of our findings were similar to another health system strengthening study by Mutiso et al*.* [38] in Makueni county*.*

Despite the WHO guidance to focus on mental health systems strengthening through continuous quality appraisal, monitoring and evaluation of existing services, there are not many efforts to review the existing mental health services in granular details in Kenya. Since health is devolved these efforts have to be carried out at county level keeping all care pathways in mind [39].

### Needed actions

Our findings highlight the need for health managers to lobby for mental health investment in order to avoid an increase in disabilities associated with untreated mental health problems. Mental health problems exist in every society and country context. However, in LMICs, the treatment, also known as the 'know do' gap, is high. This treatment gap continues to perpetuate the increasing disease burden, and thus, addressing these problems is essential to disease prevention. There is a need to map other facilities and use the conclusions to implement community mental health care policies and evidence-based mental health interventions that reduce, prevent, and promote mental health throughout the county and country. It is essential that further research investigates ways to improve the proper training for mental health care, increase health facility staffing, finance mental health programs, better provider self-care training, and enhance the supervision and appropriate referral pathways.

There are well-developed mental health plans and well-identified indicators to improve mental health care on both county and national levels. While health system strengthening approaches have been embraced by health reforms in Kenya, services are not currently providing equal and quality options to vulnerable populations. For example, little has been done to increase the budget and add human resources to combat the increasing burden of mental illnesses that mainly target adolescents.

### A case for integrated care

Through the interviews, we found that various health providers understood the meaning of integrated services and the urgency of this goal; however, they believed integration was a long-term goal rather than a short-term goal. This has also been in noted in other studies; deliberate targeting of the poor, least educated, and rural women, through the scale-up of community-level interventions, is needed to improve equity and accelerate progress. It has also been recommended that widespread implementation of key preventive and curative interventions is needed to reduce morbidity and mortality in adolescent and maternal health [40]. Integrating mental health services in the current systems would ensure that communities and the health center patients maximize service benefits.

*“Integration is when we incorporate mental health care into all service points like when someone comes to my clinic he/she is able to be screened for mental health and just the same way we are doing with TB as in every work station somewhere even from the gate the soldier can screen and know that this is a person with depression or stress or what and they may be able to assist them in a way or another. So that is the integration that-that mental health that someone-any client is able to be screened for mental health issues at every work station facility”. Reason “Yes, because previously people used to feel like mental health is-they used to stigmatize the mental health and they used to attach it to people who are mad they are in Mathari but mental health issues start from- as in with us even us the health care workers could be struggling with mental health issues and we transfer them to our clients, so it is very important when from really primary health care everyone is so conscious about their own mental health so that even as they take care of clients, they are sober and they give quality care and just to protect them from those clients from having bigger issues of mental health like depressions.”* Female Nurse

*“Integrated mental health; it is where a client comes, and all his needs are met; the psychiatric needs if he has another need like maybe he is sick or, yes if he has any other need is tackled at that point; you don't have to keep on referring here and there”* Female nurse

*“Of course it is a long term because we don't want it to like end-it is long term but it starts now [some silence] starts now [some silence] if we start with health care workers then health care workers are motivated to do it to every client then you know every clients means even those who are coming just to-they are okay but they are coming to be screened for pressure, weight, you see all those-the community groups, churches so it is a long term thing but it should start now.”* Nurse

*“This one I think it's a long-term goal because it is something that should be ongoing so it's something that's continuous because you realize that even there some people who are almost retiring; there are new employees who may be coming in the facility, there are others who are maybe transferred to other facilities; so if it is something that maybe can be done maybe as a CME or an on-job training continuously it can be good”* Female HCW

An integrated health care model would look at unified use of protocols, plans, and teamwork to care for the populations in both medical and mental service management terms. As shown in Fig. 1 of the structure of healthcare in Kenya, our mapping exercise demonstrated the urgent need to develop collaborative care models to embed mental health services with the ultimate goal of integrating mental health services. Improving guidelines and policies for mental health provision and correct information to reduce health care attitudes and discrimination related to mental health is imperative. This improvement can be achieved by training healthcare providers to assess mental disorders early on, create safe spaces in facilities, and utilize community health volunteers who can support adolescents in their respective communities. Improvements to health services are only possible if they are written and implemented at the policy level. The third level is the organization, where the political and economic are identified and sorted through decision-making systems, operating systems, or human resource systems. It is essential to invest in quality health infrastructures, such as health equipment, information, and communication technology, and transport, to allow better care and referral dimensions in primary health care. The financial, administrative, human resource, and practical clinical approaches are created to coordinate the providers’ activities at level two. Currently, the government offers free medical services in health facilities with minimal insurance coverage of mental health problems. Towards improving access and availability of quality services, efforts need to be streamlined with ongoing universal health coverage programs.

Our study’s limitations include lack of generalizability as participants were picked specifically during their free hours at work from only two facilities of Nairobi County. As opinions regarding mental health service delivery and importance may vary between people, our analysis may not represent the consensus. Also, our data analysis is purely qualitative, and our study does not include a quantitative component- presenting a limitation towards stronger external validity.

## Conclusion

This study aims to integrate mental health services in primary health care for all but especially the adolescent peripartum population in the LMIC context. Implementation has been hampered by a lack of financial and trained human resources and social barriers. Our study also underscores various mental health service improvements over the past few years, including increased services and collaboration between partner organizations.

Future research should investigate the implementation of an integrated care model to reduce the mental health treatment gap at the primary care level, specially tailored to peripartum adolescents seeking mental health care.

## Data Availability

NIMH-funded research requires the data to be made available for further studies, and this would be the case in this work. All the personal information would be de-identified, and the data put on excel sheets for research use. Some interview transcripts would be kept by the researcher and may be shared on reasonable request.

## Abbreviations

UHC: Universal Health Coverage
CHA: Community health assistant
MOH: Ministry of Health
PHC: Primary Health Care
WHO: World Health Organization
DALYs: Disability adjusted life years
SSA: Sub-Saharan Africa
YLDs: Years lived with disabilities
WHO AIMS: World Health Organization Assessment Instrument for Mental Health Systems
LMICs: Low and middle-income countries
WHO mhGAP: Mental Health Gap Action Program
CCC: Comprehensive care clinic
T.B: Tuberculosis
CDF: Constituency Development Fund
HCW: Health care workers
ANC: Antenatal clinic
PMTCT: Prevention of mother to child transmission
PESTLE: Political, economic, social, technology, legal and environment framework
HIV: Human immunodeficiency virus
HTS: HIV testing services
NCD: Non-communicable diseases
CBO: Community based organizations
ICT: Information and communications technology
CHW: Community health workers
MSF: Medecines Sans Frontieres
FIC/NIH: Fogarty International Center of the National Institutes of Health
HRH: Nairobi City County Human Resource Health Strategy
GOK: Government of Kenya
